# Cardiac and inflammatory biomarkers do not correlate with volume of heart or lung receiving radiation

**DOI:** 10.1186/s13014-014-0324-1

**Published:** 2015-01-09

**Authors:** Angera H Kuo, Marek Ancukiewicz, Kevin R Kozak, Torunn I Yock, Timothy P Padera

**Affiliations:** Department of Radiation Oncology, MGH Cancer Center, Massachusetts General Hospital and Harvard Medical School, Cox-737, 100 Blossom Street, Boston, MA 02114 USA; Current Affiliation: Institute for Stem Cell Biology and Regenerative Medicine, Stanford University School of Medicine, Stanford, CA USA; Current Affiliation: Mercy Regional Cancer Center, Janesville, WI USA

**Keywords:** Radiation, Lung cancer, Cardiotoxicity, Radiation induced pneumonitis, Biomarkers, C-reactive protein

## Abstract

**Background:**

Thoracic and cardiac irradiation increases the risk of pulmonary and cardiovascular disease. In addition, radiation, often in combination with chemotherapy, can cause treatment-related pneumonitis. Previously, we showed that the common marker for cardiac damage, troponin T, was not elevated by chemoradiation [Lung Cancer 62:351–355, 2008]. In this study, we explore whether dose-volume metrics and biomarkers for cardiac damage, inflammation or angiogenesis could identify patients receiving thoracic radiation who would later have cardiac or pulmonary complications.

**Findings:**

To this end, we quantified cardiac biomarkers including c-reactive protein (cRP) as well as a panel of angiogenic and inflammatory molecules in thirty patients who received radiation therapy to the thorax with or without concurrent chemotherapy between May 2006 and May 2007. Serum was collected at baseline, 2 weeks into radiation treatment and at the completion of radiation therapy. Heart and lung dosimetric parameters and clinical risk factors were also examined, along with the monitoring of adverse pulmonary and cardiac events during follow-up. Contrary to our hypothesis, there was no correlation between serum biomarker levels and cardiac radiation dose. Similarly there was little association between lung dose-volume metrics and inflammatory or angiogenic biomarkers. Furthermore, there was no correlation with serum biomarkers and adverse pulmonary or cardiovascular events.

**Conclusion:**

Based on these data, acute elevations in serum biomarkers of cardiac damage, inflammation or angiogenesis should not be attributed to thoracic (chemo)radiation and elevations in such biomarkers of tissue damage should be further evaluated.

## Findings

### Background and motivation

The use of radiation to treat thoracic cancers can cause treatment-related pneumonitis [1,2]. Similarly, cardiac mortality increases in patients treated with radiation to the chest [3-5]. To date, there are no validated biomarkers to predict which patients will develop these side effects. Because these patient populations tend to have extensive co-morbidities, as well as a generally poor overall prognosis, the harmful effects of thoracic irradiation do not necessarily manifest clinically in many patients. However, as survival continues to improve with better systemic therapies and more effective radiation dose delivery, greater focus is needed on the toxicities of thoracic radiotherapy.

Certain markers of inflammation (*i.e*. Interleukin-6) rise in patients that go on to develop pneumonitis [6,7], however no study has evaluated a large panel of inflammatory markers in individual patients. Furthermore, cardiac disease risk factors and symptoms overlap with those of thoracic malignancies, which might mask early signs of cardiac disease. Thus understanding how known biomarkers of cardiac damage change during thoracic radiotherapy might improve early detection of cardiac compromise and may allow real-time modification of radiation treatment to prevent further toxicity. Robust predictive biomarkers of radiation induced cardiac or pulmonary pathologies would permit identification of patients that require intense monitoring after radiation treatment. These patients may potentially benefit from earlier intervention to limit morbidity from radiation induced pathologies.

## Materials and methods

Serum samples were used from a cohort of patients previously published in [8] and summarized in Table 1. Briefly, between May 2006 and May 2007, blood was collected from 30 patients older than 18 years undergoing three-dimensional conformal or intensity modulated radiation therapy for a thoracic malignancy at Massachusetts General Hospital. Patients were excluded if they had a history of renal failure, hematocrit of less than 25% or previous thoracic radiotherapy. The study was approved by the Human Research Committee of Partners Healthcare and written informed consent was obtained from all patients. The average serum storage time at −80°C was approximately 30 months, which should maintain stability of the analytes including cRP [9]. Each patient gave three samples: the first obtained 0–8 days prior to radiation therapy (Day 0), the second following 8–10 fractions of radiation therapy (Day 10) and the third at the end of radiation therapy (Day 42). Cardiac and pulmonary dose-volume metrics were collected from approved treatment plans by a radiation oncologist and entire heart and lungs were contoured on radiation planning CT images. These data are summarized in Table 2. Adverse cardiac and pulmonary events were diagnosed clinically and recorded.

**Table 1:** **Patient characteristics (N = 30)**

**Characteristic**	**N**	**%**
Male	17	57%
Female	13	43%
Diagnosis		
Non-small cell lung cancer	18	60%
Small cell lung cancer	4	13%
Esophageal cancer	5	17%
Gastroesophageal junction cancer	2	7%
Thymic carcinoma	1	3%
Stage		
I	1	3%
II	6	20%
III	13	43%
IV	5	17%
Limited small cell lung cancer	4	13%
Recurrent	1	3%
Prior resection		
Yes	7	23%
No	23	77%
Induction chemotherapy		
Yes	7	23%
No	23	77%
Concurrent chemotherapy		
Yes	24	80%
No	6	20%

**Table 2:** **Radiation dosimetry for the heart and lungs (n = 30)**

	**Cardiac**		**Pulmonary**	
**Parameter**	**Median (range)**	**Mean ± SEM**	**Median (range)**	**Mean ± SEM**
Mean organ dose (Gy)	12.7 (0.6-41.0)	13.4 ± 1.9	14.0 (3.3-34.3)	14.1 ± 1.1
Maximum organ dose (Gy)	50.3 (1.6-73.4)	47.0 ± 3.7	57.9 (42.7-80.5)	61.3 ± 1.9
V5Gy (%)	63.4 (0–100)	55.7 ± 6.8	61.3 (10.0-92.3)	58.1 ± 4.2
V10Gy (%)	44.8 (0–100)	42.1 ± 5.9	43.5 (7.9-87.1)	42.7 ± 3.4
V15Gy (%)	31.5 (0–100)	33.2 ± 5.3	34.5 (7.1-82.2)	34.0 ± 2.8
V20Gy (%)	22.1 (0–100)	27.3 ± 4.9	26.6 (5.1-78.0)	27.2 ± 2.6
V30Gy (%)	11.9 (0–87.3)	16.9 ± 3.7	14.3 (2.4-65.3)	16.9 ± 2.2
V40Gy (%)	4.3 (0–65.0)	9.4 ± 2.7	8.3 (0.3-42.2)	10.4 ± 1.7
V50Gy (%)	0 (0–15)	2.0 ± 0.6	3.0 (0–20.0)	4.5 ± 0.9
V60Gy (%)	0 (0–3.2)	0.3 ± 0.1	0 (0–8.1)	1.8 ± 0.5
V70Gy (%)	0 (0–0.2)	0	0 (0–2.6)	0.2 ± 0.1

VXGy (%) represents the percent of the total organ volume receiving a dose of at least X Gy.

Serum samples were analyzed using multiplex ELISA from Mesoscale Discovery (Rockville, MD) and read on a Mesocsale Discovery multiplex plate reader (Sector Imager 2400). Each sample was run in duplicate on each of 3 different multiplex ELISA arrays to include the following 18 analytes: c-reactive protein (cRP), serum amyloid A (SAA), vascular cell adhesion molecule-1 (VCAM-1) and intracellular adhesion molecule-1 (ICAM-1) (Vascular Injury II kit; Mesoscale Discovery); interleukin (IL)-1β, IL-2, IL-4, IL-5, IL-6, IL-8, IL-10, IL-12, granulocyte-macrophage colony-stimulating factor (GM-CSF) and tumor necrosis factor-α (TNF-α) (Human Demonstration 10-Plex Tissue Culture Kit; Mesoscale Discovery); basic fibroblast growth factor (bFGF), placental growth factor (PlGF), vascular endothelial growth factor (VEGF) and soluble VEGF receptor-1 (sVEGFR-1) (Human Growth Factor I Kit; Mesoscale Discovery).

### Statistics

Principal component analysis (PCA) was performed to reduce the number of variables characterizing dosimetry in order to relate dosimetry to clinical events. Dosimetry is described by the mean dose to the organ (i.e. heart or lungs), the maximum dose to the organ and the percent of the organ volume that received a dose of at least 5, 10, 15, 20, 30, 40, 50, 60 and 70 Gy. The first principal component for 11 variables describing the dose to the heart (Hmax, Hmean, HV5, HV10, HV15, HV20, HV30, HV40, HV50, HV60, HV70) accounted for 61% of the variance and inversely correlated with Hmean, HV5, HV10, HV15, HV20 and HV30. The first principal component for 11 variables describing the dose to the lungs (Lmax, Lmean, LV5, LV10, LV15, LV20, LV30, LV40, LV50, LV60, LV70) accounted for 52% of the variance and inversely correlated with Lmean, LV5, LV10, LV15, LV20 and LV30. The correlation of dosimetric parameters with bioanalyte protein levels, as well as the correlation between cRP and SAA, was assessed using Kendall’s test with Benjamini-Hochberg adjustments for multiple comparisons [10]. Based on our sample, we could detect Kendall’s tau = 0.34 with a power of 80% as statistically significant with p < 0.05. Follow-up time was calculated using the “reverse Kaplan-Meier” Method described by Schemper and Smith [11]. To determine whether SAA values in individual patients increased or decreased after 10 days of radiation therapy, we used two criteria, as the baseline SAA levels in this patient population varied over 2 orders of magnitude. A rise in SAA was determined by a 50% increase in SAA from Day 0 to Day 10 as well as at least a 10 ng/ml increase in SAA. A decrease in SAA was determined by a 50% decrease in SAA from Day 0 to Day 10 as well as at least a 10 ng/ml decrease in SAA. The same criteria were used for cRP (using 10 μg/ml as the absolute cut-off).

## Results

### Patient follow-up

Extending the average follow-up time to 3.4 years of the patient cohort previously published [8], four out of the 30 patients developed pneumonitis after radiation and 12 of the 30 patients had cardiovascular events (myocardial infarction, stroke, deep vein thrombosis, atrial fibrillation, pulmonary embolism). Complications were recorded solely based on a clinical diagnosis of the event.

### Serum amyloid A (SAA) is elevated during radiation therapy

**Figure 1:**
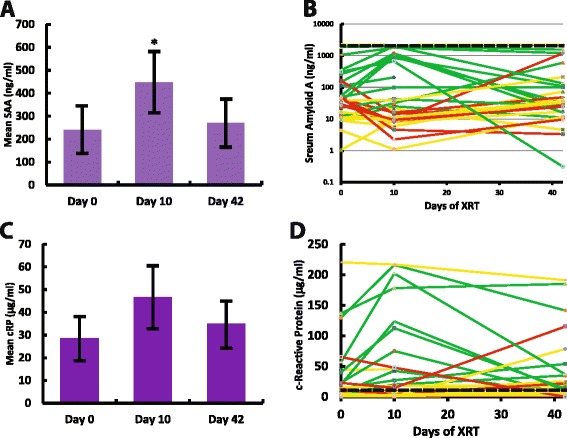
**Serum amyloid A (SAA) rises in select patients during thoracic radiation. A)** Average SAA rose during thoracic radiation and returned to pre-treatment levels after radiotherapy was complete. **B)** Individual patients showed heterogeneity in the response of SAA during thoracic radiation. Green lines show patients with increased levels of SAA after 10 days of radiotherapy. Red lines show patients with decreased levels of SAA after 10 days of radiotherapy. Yellow lines show patients with no change in SAA after 10 days of radiotherapy. Dashed line shows upper limit of normal values for SAA (2000 ng/ml). Data plotted on log scale to highlight the three orders of magnitude spread in the range of values. **C)** c-Reactive Protein (cRP) showed an elevated trend during radiation but similarly returned to pre-treatment levels after radiotherapy. **D)** Individual patients showed heterogeneity in the response of cRP during thoracic radiation. Green lines show patients with increased levels of cRP after 10 days of radiotherapy. Red lines show patients with decreased levels of cRP after 10 days of radiotherapy. Yellow lines show patients with no change in cRP after 10 days of radiotherapy. Dashed line shows upper limit of normal values for cRP (10 μg/ml). Data in (a) and (c) are plotted as mean ± SEM. *p < 0.05.

Of the 18 analytes measured in patients receiving thoracic radiation, only SAA—a protein associated with inflammatory stimuli in a variety of settings [12,13]—showed a statistically significant change during the course of therapy. SAA was elevated at Day 10 when taking the average of all 30 patients (Figure 1A). However, individual patients responded differently, with only 14 of the 30 patients seeing increased levels of SAA at Day 10, while 8 patients had decreased levels and 8 patients showed no difference (Figure 1B). The first principal component of cardiac dosimetry was related to the average SAA levels at Days 10 and 42. Similarly the mean radiation dose to the heart correlated to the average SAA levels at the same time points. Additionally, there was a significant correlation between the percent volume of the heart receiving 5 Gy and SAA levels at Days 10 and 42. However, this correlation with SAA levels was not found when the percent volumes of the heart receiving 30 or 50 Gy were tested.

**Figure 2:**
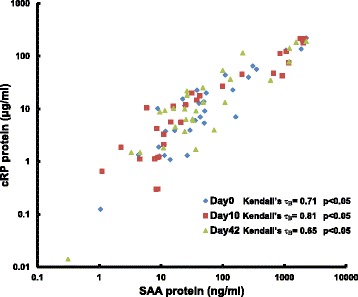
**SAA and cRP are strongly correlated.** The correlation of SAA and cRP is independent of the timing the sample was taken during the course of thoracic radiation therapy.

Our data also showed a very weak association with levels of cRP and cardiac dosimetry. 10 of the 30 patients showed increases in cRP at Day 10, where only 4 patients showed a decrease (Figures 1C,D). However, the magnitude of the increase in cRP was not large enough to detect a statistically significant difference in this population. SAA and cRP circulating protein levels positively correlated for each individual time point (Kendall’s **τ** = 0.71, 0.81 and 0.65 at Day 0, 10 and 42, respectively; p < 0.05; Figure 2), suggesting that they are mirroring the same underlying biology. Furthermore, changes from Day 0 in SAA and cRP circulating protein levels also positively correlate (Kendall’s **τ** = 0.64 and 0.63 for changes from day 0 to day 10 and 42, respectively; p < 0.05), suggesting that these proteins rise and fall together and share a common response mechanism. However from these data, SAA is a more sensitive marker of radiation dose to the heart.

Even though there was an increase in SAA levels at Day 10, the levels trended back to baseline by the end of therapy. Importantly, there was no correlation between patients with a rise in SAA and patients that eventually had cardiovascular (or pulmonary) events. Thus the rise in SAA was not predictive of a worse cardiovascular outcome in this patient population. The other 16 analytes measured did not show any correlation to cardiac dosimetry or the incidence of cardiac events (data not shown).

### Inflammatory biomarkers are not correlated with pneumonitis

It is known that the dose of radiation to the lungs is correlated with pneumonitis [1]. The data from our patient sample are consistent with this finding, as the pneumonitis incidence correlated with the first principal component of pulmonary dosimetry (p < 0.05), with higher doses corresponding to the incidence of pneumonitis. However, none of the 18 serum biomarkers correlated with lung dose-volume metrics (data not shown). Furthermore, there was no correlation between these biomarkers and the incidence of pneumonitis. Thus, based on this patient sample, these biomarkers are not robust in predicting pneumonitis in patients receiving thoracic radiation.

## Conclusions

Although our patient sample is limited, no strong candidate serum biomarker emerged, which is a necessary condition for patient stratification. Furthermore, individual patient responses varied greatly (Figures 1B,D), making a prediction in any particular patient even more challenging. Nevertheless, small rises in cRP and SAA can be expected when the heart receives radiation in the course of thoracic radiotherapy, suggesting that they are not highly specific biomarkers if a cardiac event is suspected in this setting. These patients need alternative tests if cardiac events are suspected during therapy. Surprisingly, lung radiation did not elevate the biomarkers we measured. However, even in this small study, the volume of lung and dose received correlated to the development of pneumonitis, consistent with the literature on this topic [2]. Thus, a predictive serum biomarker for pneumonitis in the acute phase remains elusive [7].

## Abbreviations

cRP: c-reactive protein
IL: Interleukin
PCA: Principal component analysis
SAA: Serum amyloid A
